# P-glycoprotein Modulates Morphine Uptake into the CNS: A Role for the Non-steroidal Anti-inflammatory Drug Diclofenac

**DOI:** 10.1371/journal.pone.0088516

**Published:** 2014-02-10

**Authors:** Lucy Sanchez-Covarrubias, Lauren M. Slosky, Brandon J. Thompson, Yifeng Zhang, Mei-Li Laracuente, Kristin M. DeMarco, Patrick T. Ronaldson, Thomas P. Davis

**Affiliations:** Department of Pharmacology, College of Medicine, University of Arizona, Tucson, Arizona, United States of America; Virginia Commonwealth University, United States of America

## Abstract

Our laboratory has previously demonstrated that peripheral inflammatory pain (PIP), induced by subcutaneous plantar injection of λ-carrageenan, results in increased expression and activity of the ATP-dependent efflux transporter P-glycoprotein (P-gp) that is endogenously expressed at the blood-brain barrier (BBB). The result of increased P-gp functional expression was a significant reduction in CNS uptake of morphine and, subsequently, reduced morphine analgesic efficacy. A major concern in the treatment of acute pain/inflammation is the potential for drug-drug interactions resulting from P-gp induction by therapeutic agents co-administered with opioids. Such effects on P-gp activity can profoundly modulate CNS distribution of opioid analgesics and alter analgesic efficacy. In this study, we examined the ability of diclofenac, a non-steroidal anti-inflammatory drug (NSAID) that is commonly administered in conjunction with the opioids during pain therapy, to alter BBB transport of morphine via P-gp and whether such changes in P-gp morphine transport could alter morphine analgesic efficacy. Administration of diclofenac reduced paw edema and thermal hyperalgesia in rats subjected to PIP, which is consistent with the known mechanism of action of this NSAID. Western blot analysis demonstrated an increase in P-gp expression in rat brain microvessels not only following PIP induction but also after diclofenac treatment alone. Additionally, *in situ* brain perfusion studies showed that both PIP and diclofenac treatment alone increased P-gp efflux activity resulting in decreased morphine brain uptake. Critically, morphine analgesia was significantly reduced in animals pretreated with diclofenac (3 h), as compared to animals administered diclofenac and morphine concurrently. These novel findings suggest that administration of diclofenac and P-gp substrate opioids during pain pharmacotherapy may result in a clinically significant drug-drug interaction.

## Introduction

The blood brain barrier (BBB) is a critical and dynamic barrier that exists between the systemic circulation and the central nervous system (CNS). Primary roles of the BBB include protection of the CNS from potentially harmful neurotoxic substances and maintenance of the homeostatic environment within the brain that is necessary for proper neuronal function. In particular, the BBB greatly limits the ability of drugs to permeate brain parenchyma and achieve efficacious concentrations. This dynamic barrier tightly regulates drug access to the CNS via two principal mechanisms: i) a physical barrier comprised of tight junction protein complexes between capillary endothelial cells that limit paracellular diffusion; and ii) a biochemical barrier characterized by endogenous transporters localized to the luminal and abluminal membranes of capillary endothelial cells and metabolizing enzymes that are expressed intracellularly [1]–[5]. BBB transporters include both influx and efflux transport proteins that play a critical role in barrier selectivity by determining what substances (i.e., drugs) are able to permeate the microvascular endothelium and access the brain.

P-glycoprotein (P-gp) is perhaps the most prominent efflux transporter expressed at the BBB. Located on the luminal and abluminal membrane surface of brain microvascular endothelium [6], P-gp's vast substrate profile renders it a formidable obstacle for effective drug delivery to the brain and efficacious treatment of CNS and non-CNS disorders such as epilepsy, HIV-1 encephalitis, Alzheimer's disease, and peripheral inflammatory pain (PIP) [7]–[10]. Known substrates of P-gp include, but are not limited to, antibiotics, calcium channel blockers, cardiac glycosides, chemotherapeutics, immunosupressants, anti-epileptics, anti-depressants, and HIV-1 protease inhibitors [11]. Additionally, previous studies have shown that opioid analgesic drugs (i.e., morphine), and opioid analgesic peptides (i.e., DPDPE), are directly extruded from brain tissue by P-gp [7], [12]–[14]. Furthermore, pathophysiological stressors can up-regulate P-gp functional expression at the BBB, which leads to an even more formidable obstacle to effective CNS drug delivery. Our laboratory has demonstrated that λ-carrageenan-induced PIP significantly increases P-gp expression at the BBB, an effect that was directly correlated with both reduced CNS morphine uptake and decreased antinociception [7]. However, the exact peripheral signal linking PIP to P-gp expression and/or activity changes at the BBB has not been clearly elucidated.

As polypharmacy becomes increasingly common, identifying drug-drug interactions involving P-gp has become critical. The ability of P-gp to interact with a myriad of structurally diverse therapeutics makes it an ideal vehicle through which ineffective drug dosing and deleterious drug-drug interactions may occur. For example, *in vivo* studies in rats with spontaneous recurrent seizures demonstrated that pharmacological inhibition of cyclooxygenase (COX)-2 signaling significantly induced P-gp expression in the brain and reduced CNS delivery of phenytoin, a known P-gp substrate [15]. Such interactions are highly probable in pharmacotherapy of pain due to utilization of multiple therapeutics in pain management regimens. For example, non-steroidal anti-inflammatory drugs (NSAIDs) such as diclofenac are frequently administered concurrently with opioids (i.e., morphine) for treatment of post-operative pain as well as for cancer pain therapy [16], [17]. Although NSAIDs have not been shown to alter P-gp mediated transport at the BBB, they have been reported to modulate P-gp in other model systems. For example, currently marketed NSAIDs including diclofenac and indomethacin were shown to increase functional expression of P-gp in human intestinal epithelial (i.e., Caco-2) cells [18]. Based on this observation, it stands to reason that NSAIDs may also affect brain-to-blood transport mediated by P-gp at the BBB, an effect that can lead to deleterious drug-drug interactions. Thus, it is vital to not only understand mechanisms regulating P-gp functional expression under pathophysiological conditions, but also those mechanisms that enable therapeutics themselves to modulate P-gp activity. Such knowledge can offer insight into how drug-drug interactions can be avoided and/or mitigated.

In the present study, we examined the effect of the commonly prescribed NSAID diclofenac on i) P-gp expression in rat brain microvessels; ii) P-gp-mediated transport of the established substrate drug morphine; and iii) morphine analgesic efficacy. All of these research objectives were evaluated *in vivo* using the naïve animals and the λ-carrageenan model of PIP.

## Materials and Methods

[^3^H]morphine (19.4 Ci/mmol) and morphine sulfate were gifts from the National Institute on Drug Abuse (NIDA) Division of Neuroscience and Behavioral Research. Rabbit polyclonal antibody against P-gp (H-241; 200 µg/mL), which recognizes amino acids 1040–1280 of P-gp derived from both mdr1a and mdr1b, was purchased from Santa Cruz Biotechnology (Santa Cruz, CA). Mouse monoclonal antibody against P-gp (C-219; 100 µg/mL), which recognizes the highly conserved amino acid sequences VQEALD (C-terminal) and VQAALD (N-terminal) on mammalian P-gp, was purchased from ID Labs (London, ON, Canada). Unless otherwise stated, all drugs and chemicals were purchased from Sigma-Aldrich (St Louis, MO).

### Animals and Treatments

The University of Arizona's Institutional Animal Care and Use Committee (IACUC) approved all experimental protocols used in this study, which conform to National Institutes of Health (NIH) guidelines. Female Sprague Dawley rats (Harlan, Indianapolis, IN) weighing 210–290 g were housed under standard 12∶12 h treatment and were provided food and water *ad libitum*. For the 1–24 h time course, rats were given a 100 µl subcutaneous (s.c) injection of either 0.9% saline or 3% λ- carrageenan into the plantar surface of the right hind paw. At 1, 3, 6, 18, and 24 h post-paw injection, animals were anesthetized using an intraperitoneal (i.p) injection of sodium pentobarbital (64.8 mg/kg; 1.0 mL/kg) and were prepared for either microvessel isolation or *in situ* brain perfusion. For studies requiring administration of diclofenac (30 mg/kg, i.p.; 1.0 mL/kg), animals were injected 15 minutes following injection of either 0.9% saline or 3% λ- carrageenan. Previous studies in our laboratory have determined that 30 mg/kg diclofenac was effective at attenuating the effects of λ-carrageenan on paw hyperalgesia and paw edema when measured 3 h post paw injection [19]. The human equivalent dose (HED) of 30 mg/kg diclofenac, is 4.8 mg/kg, which is an extremely low and non-toxic dose for humans. The therapeutic range of diclofenac in humans ranges between 100 to 200 mg/kg [20].

### Paw Edema & Hyperalgesia

Paw edema formation caused by injection of 3% λ-carrageenan was measured using a plethysmometer (model 7141, Ugo Basile, Comerio-Varese, Italy). Edema formation was measured by volume of electrolyte solution displaced by the hind paw. To ensure consistency between measurements, the ankles of each rat were marked prior the insertion of the hind paw into the plethysmometer electrolyte solution. The hind paw was inserted into the solution up to a set marked line and the paw volume was recorded. Measurements were taken at the 3 h post paw injection of either carrageenan or saline with or without diclofenac treatment (intraperitoneal). All measurements were taken in triplicate to assure precision.

Hyperalgesia was measured using the Hargreaves radiant heat method [21]. Paw withdrawal latency was measured as time (seconds) taken to remove the hind paw from the radiant heat source. Rats were habituated to plexiglass boxes on an elevated glass table for 15 minutes prior to measurements. Measurements were taken at the 3 h post paw injection of either carrageenan or saline with or without diclofenac treatment (intraperitoneal). All measurements were taken in triplicate with a 2–5 min recovery period between measurements.

### Microvessel Isolation

At appropriate time points, animals were anesthetized with sodium pentobarbital (64.8 mg/kg) and then decapitated and their brains harvested. Brains were placed in ice-cold cerebral isolation buffer (NaCl 103 mM, KCl 4.7 mM, CaCl_2_ 2.5 mM, KH_2_PO4 1.2 mM, MgSO_4_ 1.2 mM, HEPES 15 mM), pH 7, with Roche Complete™ protease inhibitor cocktail (Indianapolis, Indiana). Meninges and choroid plexus were removed, and brain tissue was homogenized in 5 mL of ice-cold homogenization buffer (NaCl 103 mM, KCl 4.7 mM, CaCl_2_ 2.5 mM, KH_2_PO4 1.2 mM, MgSO_4_ 1.2 mM, HEPES 15 mM, NaHCo_3_ 25 mM, Glucose 10 mM, Na Pyruvate 1 mM, 64 K Dextran 1 g/10 0 mL), pH 7.4, with protease inhibitor cocktail. At this time, 8 mL of cold 26% dextran was added to the homogenate and the mixture was vortexed and centrifuged at 5,800× *g* for 10 minutes. After centrifugation, the supernatant was removed and the pellet was resuspended in 5 mL of cerebral isolation buffer. At this time, 8 mL of homogenization buffer was added and the sample was then centrifuged at 5,800× *g* for 10 minutes. Following centrifugation, the supernatant was removed and the pellet was resuspended in 5 mL of homogenization buffer. The sample was then passed through a 70 µm mesh filter (Falcon, BD Biosciences, Bedford, MA). The filtrate was centrifuged at 3300× *g* for 10 min. The supernatant was discarded and pellets were either resuspended and prepared for confocal microscopy or resuspended in 6 M urea lysis buffer containing 0.1% Triton X, 10 mmol/L Tris, pH 8.0, 1 mmol/L dithiothreitol, 5 mmol/L MgCl2, 5 mmol/L EGTA, 150 mmol/L NaCl and protease inhibitor cocktail for western blot analysis. Protein concentrations were determined using bicinchoninic acid protein assay (Pierce; Rockford, IL, USA) and protein samples were stored at −20°C until used.

### Western Blot Analysis

Microvessels treated with urea lysis buffer were analyzed for expression of P-gp. Samples (10 µg/lane) were electrophoresed on a 4–12% Bis-Tris Criterion gel (Bio-rad, Hercules, CA) at 120 V for 2 h. The gel was then transferred onto a polyvinylidene difluoride (PVDF) membrane at 6 V for 30 min followed by 20 V for 3 h, while immersed in a methanol transfer buffer (192 mmol/L glycine, 25 mmol/L Tris base, 10% methanol). The membranes were incubated with SuperBlock™ blocking buffer (Bio-Rad, Hercules, CA) containing 0.05% Tween 20 at room temperature for 1 h. Membranes were then incubated with the primary anti-P-gp H-241 antibody (1∶1000) in SuperBlock™ blocking buffer containing 0.05% Tween 20 overnight at 4°C. Membranes were then washed with TBST (6×15 min) prior to a 1 h incubation with anti-mouse secondary antibody (Amersham, Piscataway, NJ, USA) diluted (1∶5000) in SuperBlock™ blocking buffer with 0.05% Tween 20. Membranes were developed and P-gp (170 kDa) was detected using enhanced chemiluminescence (ECLplus) reagent (Amersham, Piscataway, NJ, USA). Membranes were stained for total protein with Ponceau S and the optical density (OD) of each band was normalized to the total protein in each sample (i.e., loading control) according to a previously published method [22]. Ponceau S staining has long been applied for quality control of membrane transfer and is often used as an alternative to individual housekeeping/structural proteins (i.e., actin, GAPDH) in the assessment of equal loading in Western blots. Ponceau S is a fast and fully reversible stain that, when applied and quantified prior to antibody staining, has been validated as an alternative means to immunoblotting of individual specific proteins for assessment of protein loading during Western blot analysis [22]. Since experimental manipulations (i.e., drug treatments) have been shown to alter expression of housekeeping proteins (i.e., actin, GAPDH) [23] and homogenate concentrations that allow for detection of our low-abundance proteins of interest (i.e., P-gp) put high abundance loading control proteins outside the linear range of detection [24], we chose to utilize total protein as our loading control. Bands were quantitated and corrected for background using ImageJ densitometric software (Wayne Rasband, Research Services Branch, National Institute of Mental Health, Bethesda, MD). All data was normalized to saline control values that are matched to treated animals from the same experimental day and reported as percent of control (% control). Rat liver homogenate was used as a positive control for P-gp expression.

### Confocal Microscopy

Immunofluorescence of microvessels was performed as previously described [25]. Briefly, rat brain microvessels were spread onto glass microscope slides and heat-fixed for 10 min at 95°C. The slides were then immersed in 100% ethanol and blocked in 25% normal goat serum. Immunostaining was done as previously described [19], [25]. Primary antibodies for P-gp and von Willebrand factor (an endothelial cell marker) were used at dilutions of 1∶100 and 1∶500 respectively. Alexafluor 488-conjugated anti-mouse and Alexafluor 546-conjugated anti-rabbit IgG (Invitrogen Life Technologies, Carlsbad, CA) secondary antibodies were used at 1∶1000. After washing in phosphate-buffered saline (PBS), slides were treated with ProLong™ gold antifade reagent (Invitrogen Life Technologies) and imaged on a Leica SP5-II resonant scanner confocal microscope (Leica Microsystems, Buffalo Grove, IL) using a 40X/1.25 NA PL Apo oil-immersion objective lens. Filters were appropriately set to avoid bleed-through and to enhance the signal-to-noise ratio for each fluorophore. Images were obtained with a resolution of 2048×2048 and a pixel size of 0.11 µm. Semiquantitative analysis of mean fluorescence intensity of P-gp was performed according to a previously published method [25]. Microvessels were randomly chosen from rats in each treatment group and the mean fluorescence intensity was analyzed from Leica Confocal Microscope image analysis software (Leica) with the data presented as % control. Background correction settings were the same for all images acquired to ensure that data could be accurately compared between treatment groups. All slides from control and treated rats were collected and processed in parallel. Primary antibody was omitted from slides in each treatment group as a negative control.

### *In situ* Perfusion

The *in situ* brain perfusion technique was adapted from previously published methods [26], [27] and utilized as previously described by our group [7], [14], [28]. Briefly, 3 h after hind paw injection, rats were anesthetized with sodium pentobarbital (64.8 mg/kg. i.p.) prior to perfusion with [^3^H]morphine (0.5 mCi/mL; 19.4 Ci/mmol). Once anesthetized, rats were heperanized (10,000 U/kg) and carotid arteries were isolated and cannulated with silicon tubing. The perfusion medium contained a modified mammalian Ringer solution [117 mM NaCl, 4.7 mM KCl, 0.8 mM MgSO_4_.H_2_O, 24.88 mM NaHCO_3_, 1.2 mM KH_2_PO_4_, 2.5 mM CaCl_2_.6H_2_O, 10 mM D-glucose, 39 g/l dextran (MW 70,000) and 1 g/l Evans Blue] which was continuously bubbled with 95% O_2_/5% CO_2_. The Ringer solution was filtered and warmed to 37°C before arterial infusion at a rate of 3.1 mL/min. Once both carotid arteries were cannulated, [^3^H]morphine was infused into the perfusate inflow at a rate of 0.5 mL/min for 10 min, followed by a 2 min washout period in which the rat was perfused with Ringer with no radioactive tracer. For P-gp inhibitor studies, the animals were perfused with Ringer solution containing 30 µM reversin 205 only for 10 minutes. This was followed by a 10 min perfusion in the presence of both [^3^H]morphine and 30 µM reversin 205. Radiopurity of [^3^H]morphine was confirmed by HPLC coupled to radiomatic detection prior to all experiments.

At the end of the perfusion, the rat was decapitated and the brain was harvested. The meninges and choroid plexus were removed and the brain was sectioned and placed into pre-weighed vials. After weighing the vials, 1 mL of tissue solubilizer was added to each vial and allowed to dissolve the tissue for 48 h at room temperature. Once solubilization was complete, 100 ul of 30% glacial acetic acid was added to each sample to quench luminescence. Optiphase Supermix™ scintillation cocktail (1.5 mL) was then added to each sample. Triplicate samples of 100 ul aliquots of the perfusion medium were treated in the same manner as the perfused brain samples. All samples were then measured for disintegrations per minute (dpm) (1450 Liquid Scintillation and Luminescence Counter, Perkin Elmer; Waltham, MA). The ratio of the concentration of [3H] morphine in tissue (C_brain_, in dpm/g) was compared to perfusate (C_perfusate_, in dpm/mL) and expressed as a percent ratio R_brain_ = (C_brain_/C_perfusate_) x 100%.

### Antinociceptive Analysis

A warm-water (52°C) tail flick assay was used to measure the sensitivity of the tail to a noxious thermal stimulus. Specifically, this assay measured a spinally and supraspinally mediated antinociceptive response, which provided an index of BBB permeation for morphine, a peripherally administered opioid agonist [29]. Animals were gently held around the trunk, and the distal 2/3 of the tail was immersed in a 52°C constant temperature, circulating warm-water bath. The latency to tail flick or withdrawal of the tail from the water was taken as the experimental end point with a cut off of 10 sec to avoid tissue damage. Baseline tail flick values were obtained prior to treatment (i.e., 2.81±0.7 sec). After baseline measurements were recorded, animals were administrated (i.p.) either diclofenac (30 mg/kg) alone, morphine (10 mg/kg) alone, morphine (10 mg/kg) and diclofenac (30 mg/kg) concurrently or morphine (10 mg/kg) 3 h post diclofenac (30 mg/kg) treatment. Testing was performed 30, 45, 60 and 120 min post morphine administration (or post diclofenac administration in the diclofenac alone group). Raw tail flick data were converted to area under the curve (AUC) using the trapezoidal method to enable statistical comparisons between treatment groups.

### TNF-α ELISA Analysis

An enzyme-linked immunosorbant assay (ELISA) kit (Thermo Scientific, Rockford, IL, USA) was used to determine the serum concentration of TNF-α from rats treated with *λ*-carrageenan or saline in the presence or absence of diclofenac. A standard curve for TNF-α (0 to 2,500 pg/mL) was generated using recombinant rat TNF-α and the assay was performed according to the manufacturer's instructions. Absorbance was read at 450 nm using a Synergy 2 microplate spectrophotometer (Biotek Instruments Inc., Winooski, VT, USA). Absorbance was also measured at 550 nm to correct for optical imperfections in the assay plate. The concentration of serum TNF-α was expressed as pg/mL.

### Statistical Analysis

Prism software was used to perform statistical analysis. For studies measuring changes in paw edema and hyperalgesia in the presence and absence of diclofenac, data are reported as mean ± SEM from at least nine animals per experiment. For western blot analysis, data are presented as mean ± SEM from three separate experiments where each treatment group consisted of pooled microvessels from at least three animals. For confocal microscopy, data are reported as mean ± SEM from three separate experiments where each experiment analyzed pooled microvessels from at least three animals per treatment group. These analyses examined at least 50 individual microvessels per treatment group. *In situ* brain perfusion data are presented as mean ± SEM. Statistical significance was determined using one-way ANOVA, followed by Tukey's *post hoc* multiple-comparison analysis. To determine significance between treatment groups in tail flick experiments a two-way time vs. treatment ANOVA and post hoc multiple-comparison Holm-Sidak *t*-test were used. For ELISA analysis of TNF-α serum levels, data are represented as ± SEM. Statistical significance was determined using a one-way ANOVA, followed by Dunnett's multiple comparisons test. A value of *p*<0.05 was accepted as statistically significant.

## Results

### Diclofenac Attenuates the Effects of λ-Carrageenan on Paw Edema and Hyperalgesia

**Figure 1:**
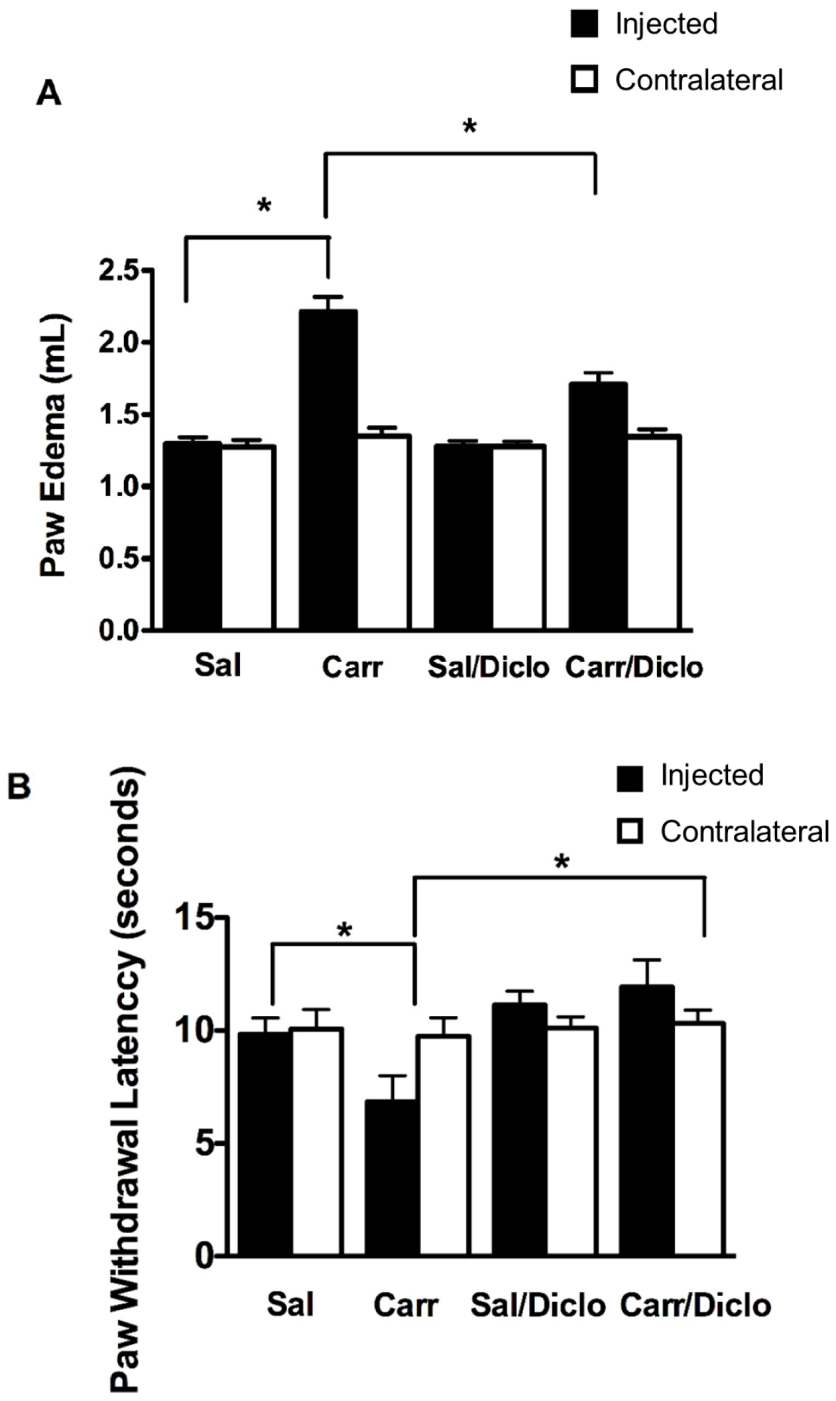
Diclofenac administration following λ-carrageenan injection attenuates PIP-induced paw edema and hyperalgesia. Animals received subcutaneous injections of saline (Sal) or λ-carrageenan (Carr) into the plantar surface of the right hind paw (injected). 15 min after paw treatment animals received saline (Sal, 1 mL/kg, i.p.) or diclofenac (Diclo, 30 mg/kg, i.p.) **A**) Administration of diclofenac resulted in a significant decrease in paw edema in λ-carrageenan treated animals 3 h post paw treatment. Edema of the untreated left hind paw (contralateral) was comparable in all treatment groups. **B**) Administration of diclofenac attenuated λ-carrageenan -induced hyperalgesia 3 h post paw treatment. Data are reported as mean ± SEM from at least nine animals per treatment group. Asterisks represent data points that are statically different as indicated (*p<0.05).

To confirm that injection of λ-carrageenan into the plantar surface of the rat hind paw evoked the expected localized inflammatory response and associated hyperalgesia [30]-[31], paw edema and thermal hyperalgesia were measured using plethysmography and the Hargreaves radiant heat test [21], respectively. 3 h post treatment animals treated with λ-carrageenan demonstrated significantly higher volumes of electrolyte solution displaced as compared to saline treated animals, confirming presence of paw edema (Figure 1A). Additionally, thermal hyperalgesia was observed in λ-carrageenan-treated animals 3 h post PIP induction (Figure 1B). A 3 h time point was chosen based on our previous work demonstrating a robust increase in paw edema and hyperalgesia at this time point [7], [32]. To confirm that formation of paw edema and onset of hyperalgesia could be attenuated by administration of an NSAID, a commonly used class of drugs used to treat pain/inflammation, we administered 30 mg/kg of diclofenac 15 minutes following λ-carrageenan paw injection. Diclofenac was administered after paw injection in order to more accurately reflect a clinical situation in which an NSAID might be used, such as treatment of pain/inflammation after an injury. As expected from previous reports [33], administration of diclofenac prevented formation of both paw edema and hyperalgesia (Figure 1). This was reflected by the observation that right hind paws (i.e., λ-carrageenan injected) of λ-carrageenan/diclofenac treated animals showed no difference in displacement of electrolyte solution (i.e., 1.27±0.36 mL) as compared to saline controls (1.30±0.42 mL) 1.27 mL. Additionally, PIP animals administered diclofenac showed a significant attenuation in paw withdrawal latency as compared to control (Figure 1B). In contrast, animals that did not receive diclofenac experienced significant paw edema and induction of hyperalgesia, an observation that further demonstrates the *in vivo* pharmacological effect of diclofenac on pain/inflammation. These behavioral and physiological data confirm that the λ-carrageenan pain model used in this study produced a reproducible and consistent inflammatory hyperalgesia specifically restricted to the right hind paw that was attenuated by diclofenac treatment.

### P-glycoprotein Expression Changes After λ-Carrageenan and Diclofenac Treatment

**Figure 2:**
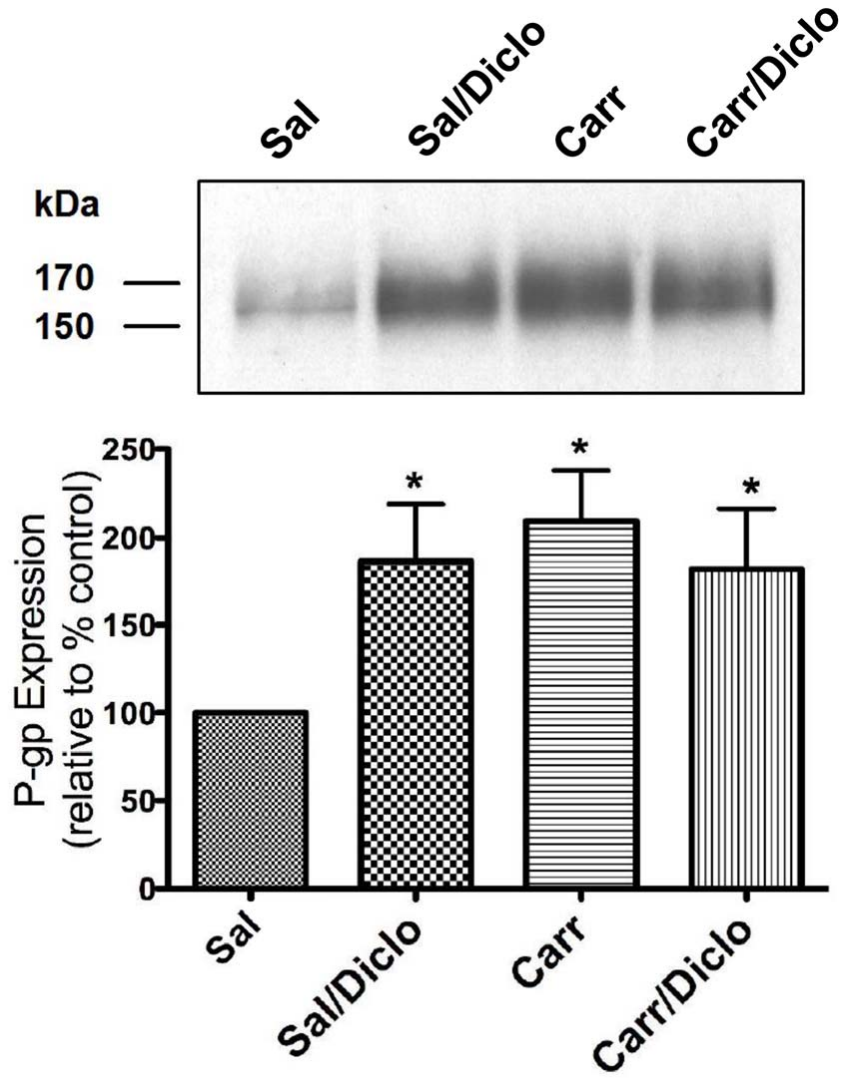
Diclofenac treatment increases P-gp expression in rat brain microvessels. Animals received subcutaneous injections of saline (Sal) or λ-carrageenan (Carr) into the plantar surface of the right hind paw. 15 min after paw treatment animals received saline (Sal, 1 mL/kg, i.p.) or diclofenac (Diclo, 30 mg/kg, i.p.). 3 h after paw treatment, animals were sacrificed and brain microvessels were isolated and prepared for Western blot analysis. Whole microvessels (10 µg) were resolved on a 10% SDS-polyacrylamide gel and transferred to a PVDF membrane. Samples were analyzed for expression of P-gp. **A**) Representative blot illustrating increased P-gp expression in animals treated with λ-carrageenan, diclofenac or both λ-carrageenan and diclofenac. **B**) Relative levels of P-gp expression in samples from **A** were determined by densitometric analysis. Results are expressed as mean ± SEM of three separate experiments (9 animals per group). Asterisks represent data points that are significantly different from saline control (*p<0.05).

Previous studies in our laboratory have shown that induction of PIP results in increased P-gp expression at the BBB [7], [32]. However, the ability of a pharmacological agent that is commonly administered for treatment of pain/inflammation (i.e., NSAIDs) to modulate P-gp expression and/or activity had not been investigated prior to the present study. Therefore, we sought to determine whether diclofenac treatment could attenuate PIP-induced increases in P-gp expression at the BBB. Consistent with our previous work, we observed increased P-gp expression in rat brain microvessels isolated from λ-carrageenan-treated animals as compared to saline controls (Figure 2). In animals subjected to PIP, administration of diclofenac did not have any effect on P-gp expression. Of particular note, diclofenac treatment in saline treated animals resulted in a significant increase (1.9-fold) in brain microvascular P-gp expression.

**Figure 3:**
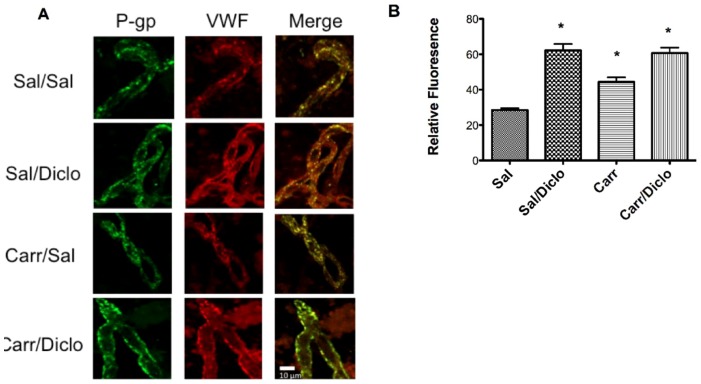
Diclofenac increases P-gp immunofluorescence. Animals received subcutaneous injections of saline (Sal) or λ-carrageenan (Carr) into the plantar surface of the right hind paw. 15 min after paw treatment animals received saline (Sal, 1 mL/kg, i.p.) or diclofenac (Diclo, 30 mg/kg, i.p.). 3 h after paw treatment animals were sacrificed and brain microvessels were isolated, plated on glass slides and fixed. Vessels were probed with mouse monoclonal anti-P-glycoprotein antibody and rabbit polyclonal antibody directed against von Willebrand factor (VWF). All slides were then incubated with appropriate Alexa Fluor-conjugated secondary antibodies. **A**. Representative immunofluorescent images from animals treated with saline paw and saline i.p. (Sal/Sal), saline paw and diclofenac i.p. (Sal/Diclo), λ-carrageenan paw and saline i.p. (Carr/Sal) or λ-carrageenan paw and diclofenac i.p. (Carr/Diclo). **B**. Semiquantitative analysis of the mean fluorescent intensity of P-gp. Microvessels isolated from treated and control animals were randomly selected for image analysis. Each bar represents the mean ± SEM of 75 microvessels from 3 separate experiments. Asterisks represent data points that are significantly different from Sal/Sal (*p<0.05).

The ability of diclofenac to increase P-gp expression at the BBB was a novel finding that had not been reported previously in the literature. Therefore, a second method was required to confirm this increase in protein expression that was observed in our western blot analyses. Confocal microscopy of fluorescent-labeled P-gp in intact microvessels was used to confirm that diclofenac itself caused an increase in P-gp at the BBB (Figure 3A). To ensure that the P-gp observed was localized to isolated brain microvessels, the vessels were also labeled with von Willebrand factor (VWF), an established endothelial cell marker. Consistent with results of our western blot analyses, vessels isolated from λ-carrageenan-treated animals showed enhanced P-gp-associated fluorescence as compared to vessels from saline control animals, indicating increased P-gp protein expression (Figure 3B). Diclofenac did not attenuate the λ-carrageenan-induced increase in P-gp. In fact, diclofenac treatment alone resulted in an increase (2.2-fold) in relative fluorescence as compared to the saline control group. This increase was comparable to the 1.9-fold increase in P-gp protein expression that was detected by western blot analysis.

### Changes in P-gp Transport Activity at the BBB – Brain Morphine Uptake

**Figure 4:**
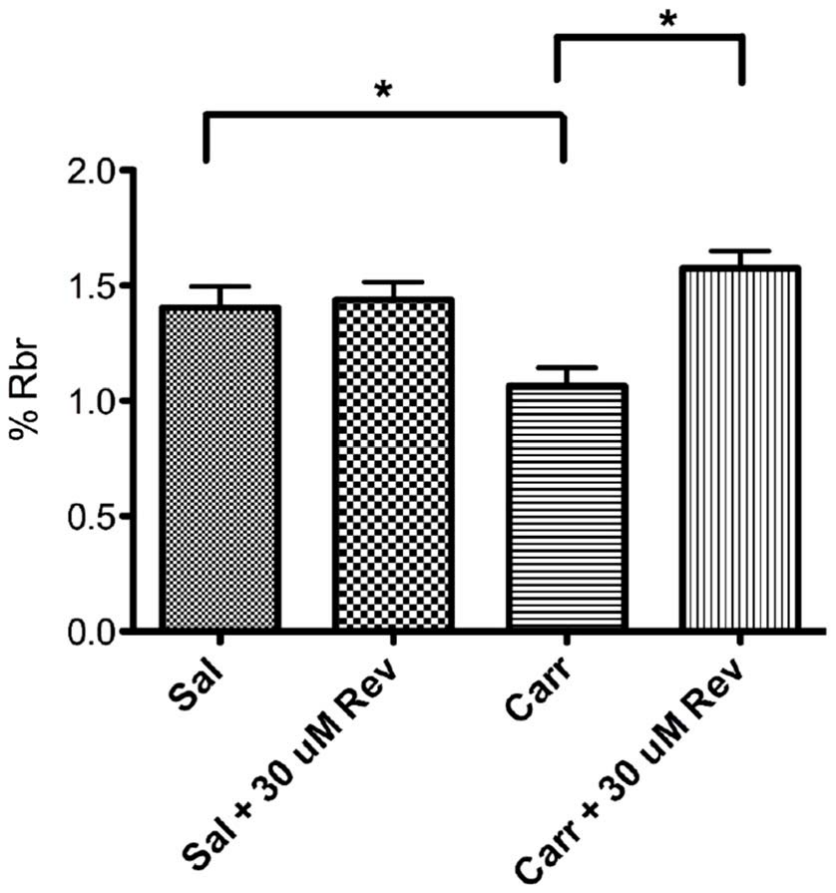
PIP increases P-gp-mediated transport of morphine at the BBB. The effect of λ-carrageenan treatment on brain uptake [^3^H]morphine (36.7 µg/rat) was determined by *in situ* brain perfusion. Animals received subcutaneous injections of saline or λ-carrageenan into the plantar surface of the right hind paw. 3 h post paw treatment animals were perfused with [^3^H]morphine (0.5 mCi/mL) for 10 min. As previously shown, λ-carrageenan treatment induced an increase in P-gp function as exemplified by decreased brain morphine uptake. P-gp specific efflux of morphine was confirmed using the P-gp selective inhibitory peptide reversin 205. Animals were perfused with reversin 205 for 10 min prior to perfusion with [^3^H]morphine. Animals were then perfused with [^3^H]morphine (.5 mCi/mL) for 10 min in the presence of reversin 205. Results are expressed as mean ± SEM of at least 6 individual animals per treatment group. Asterisks represent data points that are significantly different (*p<0.05).

Since changes in protein expression do not always correlate with modifications in transport activity, we examined whether diclofenac was also capable of altering P-gp transport activity. These experiments were conducted using *in situ* brain perfusion, a technique that enables us to measure changes in accumulation of an intact radiolabeled drug into the brain. Since morphine has been shown to be a P-gp substrate [34] and is considered to be the “opiate of choice” for pain treatment of moderate to severe pain resulting from pathophysiological conditions such as cancer [35], we chose [^3^H]morphine as the test substrate for our *in situ* brain perfusion studies. In PIP animals, a significant decrease in brain morphine uptake was observed (Figure 4). In order to confirm that the reduction in morphine uptake was due to modified P-gp transport activity, we conducted *in situ* perfusion studies in the presence of the P-gp-selective inhibitory peptide reversin 205 [36]. In the presence of reversin 205, brain morphine uptake in PIP animals was increased as compared to λ-carrageenan-treated animals that were not perfused with reversin 205. Previous studies in our laboratory have indicated that morphine remains metabolically intact throughout the course of our profusions [7]. These results indicate that BBB morphine transport is, in part, mediated by P-gp.

**Figure 5:**
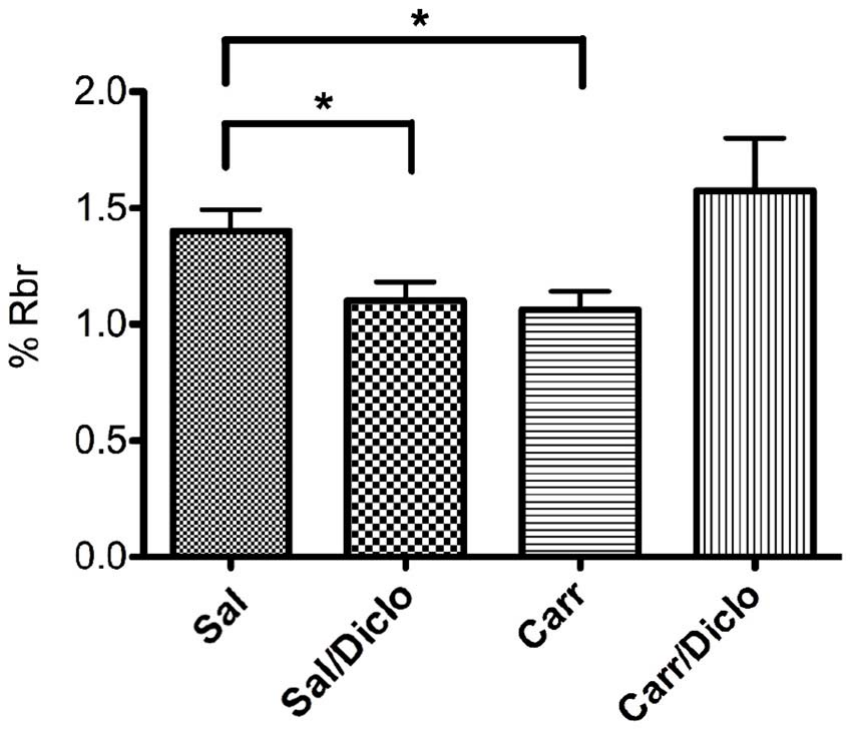
Diclofenac increases P-gp-mediated transport of morphine at the BBB. The effect of λ-carrageenan treatment on brain uptake [^3^H]morphine (36.7 µg/rat) was determined by *in situ* brain perfusion. Animals received subcutaneous injections of saline or λ-carrageenan into the plantar surface of the right hind paw. 15 min after paw treatment animals received saline (1 mL/kg, i.p.) or diclofenac (30 mg/kg, i.p.). 3 h post paw treatment animals were perfused with [^3^H]morphine (0.5 mCi/mL) for 10 min. Morphine brain accumulation was significantly decreased in animals that received saline paw injections with diclofenac i.p. injections (Sal/Diclo) and in animals that received λ-carrageenan paw injections with saline i.p. injections (Carr/Sal) as compared to saline treated (Sal/Sal) animals. Results are expressed as mean ± SEM of at least 6 individual animals per treatment group. Asterisks represent data points that are significantly different (*p<0.05).

Although diclofenac induced an increase in protein expression, it was unclear whether this NSAID could also result in an increase in P-gp mediated transport. Diclofenac-treated animals demonstrated decreased morphine uptake into the brain as compared to the saline control group, indicating increased P-gp transport activity (Figure 5). Interestingly, animals treated with both λ-carrageenan and diclofenac did not exhibit a reduction in morphine uptake, despite the fact that microvessels isolated from this group had increased P-gp expression levels (Figure 2). Taken together, these data provide evidence that the commonly prescribed NSAID diclofenac has considerable effects on P-gp expression and activity at the BBB. Furthermore, these data imply a great potential for drug-drug interactions in patients administered NSAIDs and opioids for management of pain/inflammation.

### Diclofenac Pretreatment Alters Morphine Antinociception

**Figure 6:**
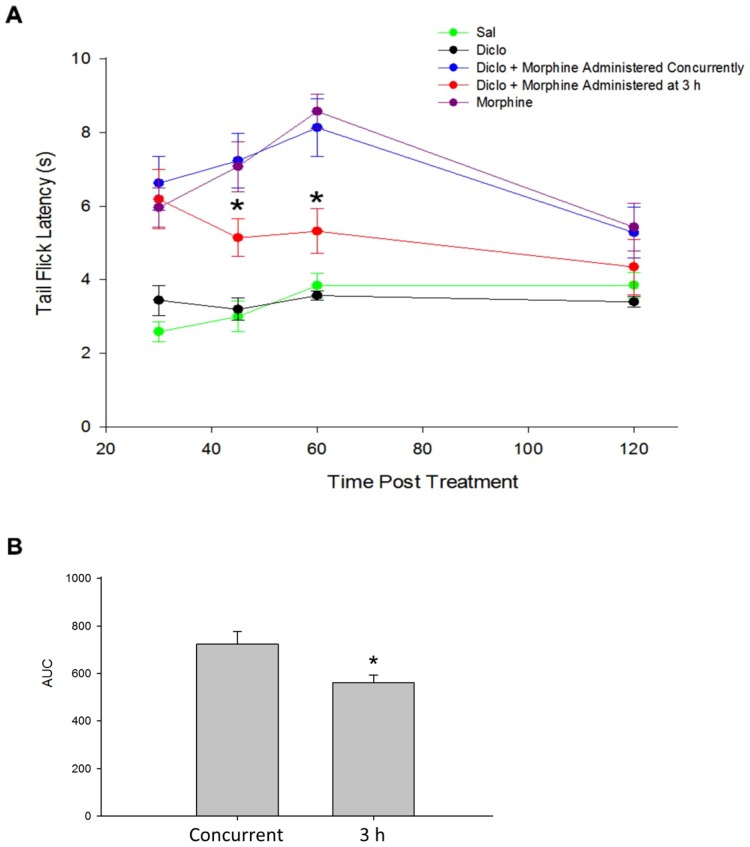
Diclofenac pretreatment alters morphine antinociception in warm-water (52°C) tail flick assay. Effect of diclofenac (Diclo, 30 mg/kg) administration on morphine (10 mg/kg) antinociception was assessed by warm-water tail flick. **A**) Time-effect curves showing an increase in tail flick latency that peaks 60 min post morphine dosing and is declining by 120 min. Two-way ANOVA indicates a decrease in morphine-induced analgesia in animals that received morphine 3 h post diclofenac treatment as compared to animals that received both morphine and diclofenac concurrently at the 45 and 60 min time points (*p<0.05). Animals receiving only saline (Sal) or diclofenac showed no change in antinociception over the 120 min time course. Results are expressed as mean ± SEM of 10 animals per treatment group. **B**) Histogram bars represent areas under the time-effect curves illustrated in **A**. Asterisks represent data points that are significantly different (*p<0.05).

As there exists an inverse relationship between morphine analgesia and P-gp expression levels [37], we speculated that altered morphine brain permeation following diclofenac treatment could result in changes in morphine analgesia efficacy. A standard warm-water (52°C) tail flick assay was used to assess morphine analgesia following diclofenac treatment. In this well-established assay for opioid-induced analgesia, an increase in tail flick latency is indicative of an increased level of analgesia. Animals were dosed with diclofenac (30 mg/kg i.p.) alone, morphine (10 mg/kg i.p.) alone, morphine and diclofenac concurrently, or morphine 3 h post diclofenac treatment. The 3 h time point were selected on the basis of Western blot data illustrating increased P-gp expression at this time points (Figure 2). Diclofenac alone had no effect on tail flick latencies (no significant difference from baseline latencies) during the 30–120 min time course (Figure 6A). Morphine alone (30–120 min) exhibited increases in tail flick latencies that were directly comparable to previously published work (Figure 6A) [38], [39]. In accordance with previous findings for morphine [39], analgesia in all animals treated with morphine and morphine with diclofenac peaked 60 min post morphine treatment (Figure 6A). Two-way ANOVA analysis showed a significant main effect for time (F(3, 49) = 5.78, p<0.001), a significant main effect for treatment (F(4, 49) = 39.83, p<0.001), and a significant time-treatment interaction (F(12, 49) = 2.16, p<0.05). Post-hoc comparisons using Holm-Sidak *t*-tests revealed differences between animals treated with morphine and diclofenac concurrently and those treated with morphine 3 h post diclofenac administration at the 45 and 60 min time points. AUC, a measure of total antinociception, was decreased in the 3 h post diclofenac group as compared to the concurrent administration group (Figure 6B). Taken together, these results suggest that animals administered morphine 3 h after diclofenac treatment, when P-gp levels are known to be elevated, experienced less analgesia than animals treated with the two drugs concurrently.

### Diclofenac Treatment Alters TNF-α Blood Serum Concentrations

**Figure 7:**
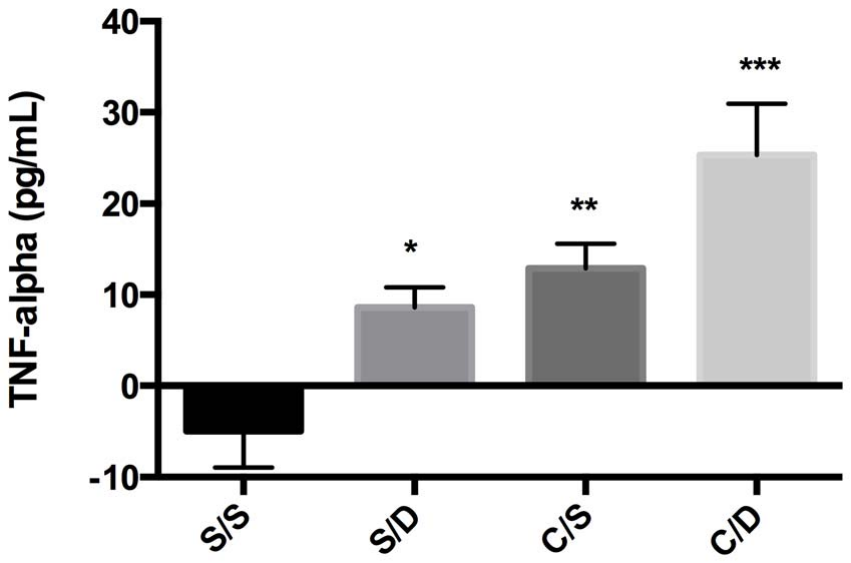
Diclofenac Treatment Alters TNF-α Blood Serum Concentrations. The effect of diclofenac (Diclo, 30 mg/kg) treatment in the presence or absence of PIP on TNF-α serum levels was assessed using a rat TNF-α ELISA. A one-way ANOVA indicates that TNF-α serum levels were significantly increased in animals treated with diclofenac both in the presence and absence of PIP (*p<0.05; ***p<0.0001). Animals treated with λ-carrageenan alone also demonstrated significantly increased TNF-α serum levels (**p<0.001). Results are expressed as mean ± SEM of 8 animals per treatment group. Asterisks represent data points that are significantly different (*p<0.05; **p<0.001, ***p<0.0001).

Several studies have demonstrated that TNF-α signaling plays a role in the regulation of P-gp expressed at the blood brain barrier [40], [41]. Additionally, diclofenac has been shown to increase TNF-α in whole blood [42]. Therefore we hypothesized that the diclofenac-induced changes in P-gp expression may be the result of changes in circulating TNF-α levels. ELISA analysis was performed on serum samples from rats receiving a paw injection of either saline of λ-carrageenan in the presence or absence of diclofenac in order to detect changes in TNF-α serum concentrations (Figure 7). In control animals, a negative value for TNF- α serum levels was calculated, which suggests that basal TNF- α concentrations are below the level of detection of this ELISA assay. Animals given a paw injection of saline and treated with diclofenac showed a significant increase in TNF-α serum levels (2.74 fold; p<0.05) compared to controls. Animals treated with λ-carrageenan and diclofenac showed a 6.13 fold increase in TNF-α serum levels (p<0.01). Serum levels in animals treated with λ-carrageenan alone showed a 3.62 fold increase compared to saline control animals (p<0.0001). These results suggest that TNF-α signaling may be involved in diclofenac-induced upregulation of P-gp functional expression at the BBB.

## Discussion

In the clinic, it is well established that combination of diclofenac with morphine in pharmacological pain management regimens results in an “opioid sparing” effect characterized by achievement of effective analgesia with lower morphine doses [43]–[46]. However, unexpected effects resulting from this particular drug combination have also been reported. For example, pre-emptive diclofenac resulted in favorable analgesia immediately following spinal surgery but those patients that received diclofenac also required a greater quantity of analgesic drugs at significantly higher doses during the post-operative period than patients who received only a continuous infusion of morphine [47]. More recently, administration of diclofenac was shown to provide minimal enhancement in pain relief in patients that were treated with opioids for non-cancer chronic lower back pain even when diclofenac was infused at the same time as morphine [48]. These clinical observations underscore the importance of our present study, which was designed to establish a mechanistic basis for drug-drug interactions between NSAIDs and opioid analgesic drugs. We propose that such drug-drug interactions likely involve changes in endogenous drug transport processes at the BBB. Since optimal pharmacotherapy with opioids requires that these drugs access the CNS [38], [49], any change in drug transport mechanisms at the BBB can potentially have dramatic effects on opioid analgesic efficacy [50]. One transporter that plays a critical role in determining CNS drug penetration is P-gp, an efflux transporter that is functionally expressed at the BBB and is known to be involved in brain-to-blood transport of morphine. Changes in P-gp protein expression and/or transport activity have been observed in various pathophysiological conditions including epilepsy, HIV-1 encephalitis, Alzheimer's disease, and PIP [7]–[10]. For example, our laboratory has shown that PIP results in increased P-gp protein expression at the BBB that was accompanied by decreased brain morphine uptake and decreased antinociception [7]. Additionally, altered P-gp functional expression in response to administration of pharmacological agents has been previously shown [7], [51], [52], [18]. For example, *in vivo* administration of rifampin, a bactericidal antibiotic, resulted in an increase in P-gp functional expression in mouse brain capillaries [51]. This increased P-gp activity was associated with decreased efficacy of methadone, an established P-gp substrate and CNS-acting synthetic opioid used to treat moderate to severe pain, as well as opioid addiction [51]. Using human intestinal epithelial cells, Takara and colleagues showed that NSAIDs such as diclofenac and indomethacin induces expression of MDR1 mRNA in Caco-2 cells [18]. Our present study expands upon this knowledge by examining, for the first time, the effect of a low and non-toxic human equivalent dose of diclofenac on P-gp-mediated transport of morphine at the *in vivo* BBB. Furthermore, our present study is highly innovative because we examine this effect in both control animals and in animals subjected to PIP.

Administration of diclofenac attenuated the hyperalgesic response evoked by λ-carrageenan. Diclofenac treatment also mitigated the onset of paw edema caused by PIP. These data indicate that diclofenac effectively functioned as an NSAID, reducing both pain and inflammation in our *in vivo* PIP model. These results agree with data from previous studies demonstrating the anti-inflammatory and antinociceptive effects of diclofenac [20]. Despite effective resolution of pain/inflammation, diclofenac treatment did not reverse PIP-induced increases in P-gp expression in cerebral microvessels. Interestingly, administration of diclofenac alone, in the absence of PIP, resulted in an increase in both P-gp protein as measured by Western blot analysis and confocal microscopy. Critically, the increase in protein expression, without a painful and/or inflammatory stimulus, was accompanied by an increase in P-gp efflux activity. This increase in activity was functionally demonstrated by a decrease in [^3^H]morphine uptake into the brain and correlated to a decrease in morphine analgesia. Although PIP has been shown to affect BBB paracellular permeability via disruption of TJ proteins, changes in morphine uptake into the brain are not likely to be the result of changes in paracellular transport. The physiochemical properties of morphine indicate that it traverses the BBB via a transcellular, not a paracellular pathway [53]. The role of P-gp in the observed change in morphine brain uptake was confirmed by use of the P-gp selective inhibitor reversin 205 [37], which effectively reserved the λ-carrageenan-induced decrease in morphine brain accumulation. Morphine uptake into the brain is governed by three processes: passive diffusion, P-gp-mediated efflux and low-capacity active influx [54]. Since morphine's passive permeability is quite low, active uptake processes for morphine are a critical determinant of morphine brain delivery. At low concentrations (i.e., 36.7 µg in present study), this active uptake process can make it appear that morphine delivery to the CNS is insensitive to P-gp inhibitors [54]. This explains why one could note that reversin 205 did not alter morphine uptake in saline control animals. However, processes that induced P-gp functional expression can make low morphine concentrations (i.e., 36.7 µg in present study) sensitive to P-gp inhibition, as we have observed here in the present study. Taken together with the results of our present transport studies, these data imply that changes in morphine uptake are the result of P-gp modulation by PIP.

Several studies have implicated P-gp in limiting brain entry and analgesic efficacy of several clinically used opioids. Mdr1a knockout mice demonstrated increased cerebral concentrations of morphine and enhanced morphine analgesic efficacy as compared to wild-type mice [55]. Additionally, ineffective morphine analgesia due to increased P-gp efflux activity has been observed under conditions of PIP [7]. Although we are the first laboratory to demonstrate modulation of P-gp functional expression at the *in vivo* BBB by diclofenac, previous studies have demonstrated the ability of NSAIDs to modulate functional expression of other transporters expressed at the BBB. Using the brain efflux index method, ibuprofen and ketoprofen were shown to reduce prostaglandin E2 transport mediated by multidrug resistance protein 4 at the murine BBB [18]. The observations published in the study by Akanuma and colleagues [18] points to the potential of NSAIDs to alter BBB transporter expression and/or activity. More studies are needed in order to determine if the P-gp-mediated reduction of morphine uptake into the CNS is diclofenac-specific or whether similar observations may be seen with the use of other NSAIDs. Currently there is no evidence in the literature that other NSAIDs can interfere with the analgesic effects of opioids via the upregulation of the functional expression of P-gp, thus highlighting the novel nature of our findings.

There is currently considerable debate on the clinical significance of P-gp induction at the BBB, particularly in regards to the potential for P-gp-mediated drug-drug interactions. To this end, the International Transporter Consortium (ITC) has recently released a position statement on the role of P-gp mediated efflux transport in determining permeation of currently marketed drugs at the human BBB and the potential for P-gp-mediated drug-drug interactions at the level of the BBB [56]. In this paper, Kalvass and colleagues state that P-gp cannot be induced at the BBB in “genetically unmodified” rodents [56]. Furthermore, the ITC goes on to postulate that the maximal *in vivo* induction of P-gp at the BBB that has been reported in the current body of literature (i.e., <2-fold) will not alter pharmacokinetics of currently marketed drugs to a degree that is sufficient to cause a detectable pharmacodynamic effect in the brain [56]. Therefore, the ITC concluded that P-gp induction at the *in vivo* BBB is extremely weak and unlikely to contribute appreciably to drug-drug interactions. In light of data obtained in our present study, we disagree with this assessment of the relationship between P-gp induction at the BBB and modified drug efficacy. One strength of our present study is that we assessed whether altered morphine brain permeation resulting from a maximal P-gp increase at the BBB of 2.2-fold would result in modified morphine analgesic efficacy using a standard warm-water (52°C) tail flick assay. We measured differences in morphine analgesia in two experimental groups: i) animals administered morphine and diclofenac concurrently, ii) animals administered morphine 3 h post diclofenac treatment when P-gp is known to be upregulated (Figure 2). In accordance with P-gp expression and transport data illustrating increased P-gp functional expression after diclofenac treatment (3 h), tail flick latency scores (in sec) showed a significant decrease in morphine-induced analgesia in animals that received morphine 3 h after treatment with a clinically relevant diclofenac dose, as compared to those that received the two drugs concurrently. Such data refute the ITC position that there is little-to-no potential for significant drug-drug interactions involving P-gp at the BBB due to the small degree of P-gp induction that has been reported in *in vivo* model systems. Furthermore, these data demonstrate that timing of diclofenac dosing is a critical determinant of outcome (i.e. effective morphine analgesia). The temporal profile of BBB effects (i.e., P-gp upregulation) in response to diclofenac treatment matches those of BBB drug permeation and behavior. In our study, multiple endpoints were used to assess the effect of diclofenac on functional P-gp expression at the BBB. These diverse endpoints, including P-gp expression levels, morphine brain permeation, and morphine analgesia, all corroborate a diclofenac-induced increase in P-gp functional expression at the BBB and corresponding decrease in P-gp substrate brain permeation and efficacy. Specifically, 3 h post-treatment with a low and non-toxic human equivalent dose of diclofenac, there is a modest increase in P-gp expression (up to 2.2-fold), as detected in isolated brain microvessels, and corresponding decreases in morphine brain permeation and morphine-induced antinociception. Such an effect on morphine analgesia is absent when diclofenac and morphine are administered concurrently (Figure 7). The importance of the timing of adjuvant drug administration with morphine has previously been demonstrated. It has been found, for example, that dexamethasone attenuates morphine analgesia in mice when administered 2 h prior to morphine dosing but not when administered only 10 min prior to morphine [57].

The restriction of opioid transport by P-gp at the BBB can result in ineffective pain relief, an observation that has important clinical implications for the use of diclofenac and morphine together in pain management. It is important to consider that clinical benefits, such as a reduced need for opioids in pain management regimens, may also arise from NSAID-opioid interactions at the level of P-gp [50]. For example, Mogadam and colleagues recently reported decreased post-operative consumption of meperidine, an opioid analgesic, in patients that were administered diclofenac prior to surgery [58]. *In vitro* studies have suggested that meperidine is a P-gp transport substrate [59]. Although studies in mdr1a knockout mice showed no difference in meperidine antinociception [60], a direct *in vivo* analysis of P-gp-mediated meperidine transport has not been undertaken. Furthermore, this study measured analgesic efficacy using only tail-pinch, a technique that may not have enough sensitivity to detect differences in meperidine analgesia in mice with differing P-gp activity at the BBB. When taken in the context of the study by Mogadam and colleagues (2012), our data suggest that modified opioid analgesic efficacy and/or reduced need for opioids in pain management regimens may reflect changes in CNS opioid delivery induced by NSAIDs (i.e., diclofenac).

Diclofenac's mechanism for regulating P-gp functional expression is unclear and the subject of active investigation in our laboratory. Data clearly demonstrated that diclofenac functioned as an NSAID in these studies, alleviating both thermal sensitivity and paw edema due to PIP (Figure 2). Despite effective analgesic and anti-inflammatory activity diclofenac was unable to attenuate the increase in P-gp expression observed following induction of PIP. Interestingly, diclofenac treatment did block the λ-carrageenan-induced increase in P-gp transport activity, as assessed via *in situ* brain perfusion (Figure 5). This discrepancy between P-gp expression and functional data highlights the complexity of P-gp regulation under both PIP and diclofenac treatment. The data point to modification of multiple, possibly opposing, P-gp regulatory pathways by PIP and diclofenac treatment. The analgesic properties of NSAIDs are linked to their ability to prevent prostaglandin production through COX inhibition. COX signaling may be involved in the regulation of P-gp under diclofenac treatment. Recent studies have demonstrated the ability of COX-2 to modulate mrd1a expression and P-gp protein expression at the rodent BBB and COX-2 and its prostaglandin products misoprostol and iloprost to alter MDR1 expression in a human brain endothelial cell line [61]. Another possible mechanism through which diclofenac modulates P-gp functional expression is by shifting the balance between COX-mediated prostaglandin production and lipoxygenase-mediated production of leukotrienes. Of interest, leukotrienes have been found to be nuclear receptor ligands [62] and the induction of mdr1a and P-gp expression at the rodent BBB by nuclear receptor activation is well established [52], [63]–[65]. The finding that COX inhibition is mediating diclofenac's effect on P-gp functional expression would have broad implications for the concomitant use of NSAIDs and P-gp substrate drugs, such as morphine. Additionally, induction of P-gp following diclofenac treatment may also be attributable to diclofenac-induced changes in cytokines such as TNF-α. Diclofenac has been found to increase TNF-α in whole blood and increased levels of TNF-α have been linked to increased P-gp functional expression at the BBB [40], [41], [51]. Indeed, studies in our own laboratory confirm these results, revealing significantly higher serum levels of TNF-α in animals treated with diclofenac in the presence and absence of λ-carrageenan-induced PIP (Figure 7). In naïve and λ-carrageenan-treated animals diclofenac increased serum TNF-alpha levels 2.74-fold and 6.13-fold, respectively, 3 hours post treatment. A thorough investigation is needed to determine whether this pro-inflammatory cytokine plays a role in the regulation of P-gp under diclofenac treatment. The contribution of TNF-α signaling to the observed increase in P-gp functional expression is the subject of ongoing studies in our laboratory.

In the current study we demonstrate, for the first time, significant diclofenac-induced changes in P-gp functional expression *in vivo* at the rodent BBB. Our data expose the potential for a drug-drug interaction involving NSAIDs (i.e., diclofenac) and P-gp substrate opioid analgesic drugs, such as morphine. Such interactions may result in inefficient pain management with opioids and/or potentially harmful adverse drug events.
